# Initiation but no execution - modulation of peripheral blood lymphocyte apoptosis in rheumatoid arthritis - a potential role for heat shock protein 70

**DOI:** 10.1186/1476-9255-8-30

**Published:** 2011-11-03

**Authors:** Devapregasan Moodley, Girish M Mody, Anil A Chuturgoon

**Affiliations:** 1 Discipline of Medical Biochemistry, Faculty of Health Sciences, University of KwaZulu-Natal, Private Bag 7, Congella, 4013, Durban, South Africa; 2 Department of Rheumatology, Nelson R Mandela School of Medicine, University of KwaZulu-Natal, Private Bag 7, Congella, 4013, Durban, South Africa

**Keywords:** Apoptosis, Lymphocyte, Rheumatoid arthritis, Heat shock protein

## Abstract

**Background:**

Rheumatoid arthritis (RA) is a chronic autoimmune disease, which causes synovial damage. Persistence of lymphocyte infiltrates in the rheumatoid synovium has been attributed to abnormal apoptosis. While not comprehensively investigated, perturbations in peripheral blood lymphocyte (PBL) apoptosis may also be involved in perpetuation of autoimmune processes in RA.

**Methods:**

We investigated total, CD4+ and CD19+ PBL apoptosis in our study cohort by monitoring the translocation of phosphatidylserine using the Annexin-V assay. To examine the role of death receptor mediated apoptosis as well as activation-induced-cell-death (AICD), PBLs were labeled with CD95/Fas and CD69 markers and enumerated by flow cytometry. Proteolytic activity of initiator and executioner caspases was determined by luminometry. DNA fragmentation assays were used to examine whether apoptotic signals were transduced to the nucleus. Quantitative PCR arrays were used to investigate apoptotic pathways associated with RA-PBLs. Since heat-shock-protein-70 (HSP70) is an inducible protein which modulates apoptotic signals, we determined HSP70 levels by intra-cellular flow cytometry and western blots.

**Results:**

The RA-PBLs showed signs of elevated apoptosis whilst in circulation. These include increases in the loss of plasma membrane asymmetry, indicated by increased externalization of phosphatidylserine (especially in B-lymphocytes). RA-PBLs showed a bias to CD95/Fas mediated apoptotic pathways, but low levels of the CD69 marker suggested that this was not associated with immune activation. Although downstream markers of apoptosis such as caspase-3/7 activity, were increased, no DNA fragmentation was observed in RA-PBLs. Interestingly, elevated levels of apoptosis did not correlate with absolute lymphocyte counts in RA patients. Levels of HSP70 were highly elevated in RA-PBLs compared to controls.

**Conclusion:**

The results suggest that while apoptosis may be initiated in RA-PBLs, they may lack commitment to fully executing the apoptotic program. This may be related to inhibition on apoptotic transduction by HSP70. This study provides evidence that abnormalities in RA-PBLs apoptosis may occur whilst still in circulation and may contribute to pathogenesis of the disease.

## Introduction

Rheumatoid arthritis (RA) is an autoimmune disease characterized by chronic joint inflammation, synovial hyperplasia and bone erosion. In RA, the synovial micro-environment is considerably altered due to secretion of pro-inflammatory cytokines and subsequent infiltration of inflammatory cells. This facilitates pannus tissue formation and invasive growth of synovial tissue into articular cartilage and bone, with eventual joint destruction [1].

Bone and cartilage erosion occur during the natural progression of RA as a result of subtle underlying abnormalities in immune regulation and function. Accumulation and persistence of the lymphocyte infiltrate in the rheumatoid synovium are characteristic features of the disease [2]. In normal inflammatory responses, lymphocytes are eliminated, upon cessation of function, by initiation of apoptotic cascades [3]. Apoptosis is the major mechanism of programmed cell death and is necessary for regulation of tissue growth and homeostasis. In particular, the immune system relies heavily on apoptosis to ameliorate inflammation in order to prevent misdirected damage to normal tissue [4].

Several lines of evidence in RA suggest that malfunctions in apoptosis are responsible not only for the persistence of synovial lymphocytes, but also for the invasive nature of fibroblast-like-synoviocytes (FLS) [5,6]. Interactions between these cell types either through cellular contact or by secretion of soluble factors contribute to impaired apoptosis and chronic inflammation of the synovial membrane [7].

In murine models of proteoglycan induced arthritis, T-lymphocyte apoptosis was shown to be defective despite high expression levels of CD95/Fas and was related to impaired downstream CD95/Fas signaling pathways [8]. Elevated levels of anti-apoptotic Bcl-2 proteins conferred resistance to CD95/Fas-induced apoptosis in CD4^+ ^T-lymphocytes from RA patients [9]. Furthermore, it was shown that RA-FLS synthesize high quantities of stromal-cell-derived-factor-1α (SDF1α), a ligand for lymphocyte chemokine-receptor-4 (CXCR4), which induces migration of CD4^+ ^T-lymphocytes to the synovium. Interestingly, SDF1α also inhibits T-lymphocyte apoptosis by interfering with mitogen-activated-protein-kinase (MAPK) pathways [10].

In addition to T-lymphocytes, there is growing interest in B-lymphocyte biology within the context of autoimmunity [11]. The recent success of anti-B-lymphocyte therapies support the notion that breakdown of normal B-lymphocyte function contributes to the pathogenesis of RA [12]. Indeed, there is accumulating evidence for impaired B-lymphocyte apoptosis in the rheumatoid synovium [13]. B-lymphocytes are enriched in the RA synovial membrane and are bound to FLS, which act as follicular dendritic cells [14]. In co-culture with RA synovial stromal cells, B-lymphocytes up-regulate expression of Bcl-xL, which inhibits mitochondrial pro-apoptotic signals [15]. Inhibition of B-lymphocyte apoptosis by FLS was shown to occur in a cell-contact dependant manner via vascular-cell-adhesion-molecule-1 (VCAM1) [16]. These data suggest that cell-contact interactions contribute to the pathophysiology ultimately leading to destruction of the rheumatoid synovium.

While synovial joints are the primary sites of inflammation in RA, there is a significant, but poorly understood systemic inflammatory component of the disease. Immuno-pathologies in RA are not limited to synovium sensitive cells but also involve the majority of circulating peripheral lymphocytes. Zhang *et al *(2001) proposed that defective apoptosis may lead to accumulation of T-lymphocytes in peripheral circulation [8]. It is likely that this may perpetuate the often elusive systemic complications of RA. There is compelling evidence to show that dysregulation of PBL apoptosis is critical in the pathogenesis of various systemic autoimmune diseases such as systemic lupus erythematosus (SLE), Sjögrens syndrome and systemic sclerosis [17,18]. Apoptosis in RA-PBLs however, has not been comprehensively investigated. Defects in RA-PBL apoptosis could clearly underlie some of the characteristic immunologic phenomena seen in RA patients.

Despite an unclear delineation of their specific roles, stress-response proteins, specifically heat shock proteins (HSPs), have been repeatedly implicated as key participators in the pathogenesis of RA [19]. Interestingly, numerous mechanisms of HSP-mediated inhibition of cell death have been described [20,21]. Since RA-PBLs exist in a milieu of inflammatory mediators such as tumor-necrosis-factor-α (TNFα) and C-reactive protein (CRP) [22], it is likely that exacerbated stress responses may interfere with transmission of apoptotic signals.

It is generally accepted that the autoimmune manifestations of RA are due, in part, to impaired lymphocyte apoptosis. Whether these defects are related to failures in executing the apoptotic program at inflammatory sites, or as a result of inherent defects in lymphocyte apoptotic machinery prior to recruitment to these sites remain to be elucidated. The apoptotic status of circulating lymphocytes in RA may be a useful indicator of underlying pathological processes or disease activity. A better understanding of PBL biology, and indeed PBL apoptosis, may provide clues to how immunological tolerance is breached in RA. Consequently, in this study we investigated whether PBL apoptosis was impaired in RA patients directly *ex vivo *and whether there was an association with HSP70.

We report that PBL showed early signs of elevated apoptosis in our patient cohort and seemed to be associated with CD95/Fas, but is not necessarily related to lymphocyte activation. Interestingly, despite the significantly high levels of PBL apoptosis measured in patients, absolute lymphocyte counts remained high. In addition, we did not observe any downstream markers of apoptosis such as DNA fragmentation in patients. Our data also shows elevated levels of heat-shock-protein-70 (HSP70), which correlated with phosphatidylserine externalization. HSP70 is a known modulator of apoptosis and may interfere with transmission of apoptotic cascades in RA-PBLs. We suggest that abnormal control of PBL apoptosis may contribute to autoreactivity in persons who develop RA.

## Materials and methods

### Patient recruitment

Fifty South African black RA patients attending the Rheumatology clinic at Inkosi Albert Luthuli Central Hospital (Durban, South Africa) were recruited into the study. All patients (mean age: 50.7 years, range 18-75 years; mean duration of disease: 13.3 ± 9.5 years) fulfilled the American College of Rheumatology (ACR) criteria for RA [23] and were on disease-modifying antirheumatic drug (DMARD) treatments. Patient recruitment commenced following institutional ethical approval (H109/04) and informed consent was obtained for each patient. The patients reported no recent/chronic infection or history of other chronic inflammatory diseases. Clinical and laboratory parameters (mean number of swollen joints and tender joints; erythrocyte sedimentation rate, ESR; C-reactive protein, CRP; absolute lymphocyte counts) were recorded for all the patients, (Table 1). An equal number of healthy race matched control samples were sourced from the South African National Blood Services following routine screening. Both control and patient groups had a female to male ratio of 6:1. Control samples were extracted and assayed in the same manner as patient samples to ensure comparability.

**Table 1:** Summary of clinical and laboratory parameters measured in RA patients

Parameter	Result (Mean ± SD)
Mean number of swollen joints	12 ± 6.7
Mean number of tender joints	13 ± 8.7
ESR	42.3 ± 28.5 mm/hr
CRP	19.59 ± 20.5 mg/ml
Mean lymphocyte count	6.9 ± 2.7 × 10^9 ^/l

Patients Treatments:	
Patients on MTX	13
Patients on MTX + steroids	29
Patients on other DMARDs	8

Disease Duration:	
< 10 years	22
> 10 years	28

### Peripheral blood mononuclear cell (PBL) preparation

Buffy coats containing PBL were extracted from heparinized whole blood by differential centrifugation. Briefly, 5 ml whole blood collected from each subject, was layered onto equivolume Histopaque 1077 (Sigma, Germany) in 15 ml polypropylene tubes. Layered blood was then centrifuged at 400 × g for 30 minutes. Buffy coats were aspirated into new polypropylene tubes and washed twice in phosphate buffered saline (PBS) (400 × g, 10 minutes). Cell density was adjusted to 1 × 10^6 ^cells/ml after exclusion of dead cells with trypan blue. Sample preparation and subsequent apoptosis assays were complete within 3-4hrs after drawing of blood from study participants.

### Detection of phosphatidylserine on outer membrane of peripheral lymphocytes

The annexin-V-FITC apoptosis detection kit (Roche, Germany) was used to label apoptotic PBL with translocated phosphatidylserine residues on the outer plasma membrane. In addition to annexin-V-FITC, the kit contained propidium iodide (PI) which was used to monitor late stage apoptosis and necrotic cell death. The assay procedure was as per manufacturer's instructions. Briefly, annexin-V-FITC labeling solution (100 μl) was added to 1 × 10^6 ^PBL in cytometry tubes and allowed to incubate for 15 minutes in the dark at room temperature (RT). Following incubation, two separate aliquots of the annexin-labeled PBL were prepared in order to assess apoptosis in B- and T-lymphocyte sub-populations. Allophycocyanin (APC)-labeled anti-CD4 and anti-CD19 (Pharmingen, USA) was added (5 μl) to the respective PBL aliquots 10 minutes prior to enumeration by flow cytometry.

### Detection of CD95/Fas and activation marker CD69 on peripheral lymphocytes

Aliquots of approximately 1 × 10^5 ^PBL were transferred into cytometry tubes containing monoclonal mouse anti-CD95/Fas (1:100) (Sigma, Germany). The mixture was allowed to react for 20 minutes and PBL were thereafter washed in PBS (400 × g, 10 minutes). To detect Fas-positive PBL by flow cytometry, rabbit anti-mouse APC-labeled secondary antibody (Sigma, Germany) was added to cells at a final dilution of 1:1000 and allowed to react for 15 minutes. In order to determine the activation status of circulating lymphocytes in RA patients, 1 × 10^5 ^PBL were incubated with 10 μl fluorescein-isothiocyanate (FITC) labeled anti-CD69 (BD Biosciences) for 15 minutes prior to analysis by flow cytometry.

### Intra-cellular detection of heat shock protein 70 (HSP70)

For each sample, 1 × 10^5 ^PBL were transferred into cytometry tubes. Cells were then fixed (100 μl Caltag reagent A fixative medium; Caltag Laboratories, USA) for 15 minutes at RT. After fixation, PBL were washed in PBS supplemented with 0.1% sodium azide and 5% fetal bovine serum (300 × g, 5 minutes). Thereafter, PBL were re-suspended in permeabilization medium (100 μl, Caltag reagent B permeabilization medium; Caltag Laboratories, USA) containing monoclonal mouse anti-HSP70 (1:1, 000; Pharmingen, USA) for 30 minutes. Following permeabilization and incubation with primary antibody, PBL were washed twice as previously described. Samples were then incubated with APC-conjugated rabbit anti-mouse secondary antibody (1:10, 000; Pharmingen, USA) for 20 minutes at RT in the dark. After an additional wash step, labeled PBL were re-suspended in sheath fluid for detection by flow cytometry.

### Flow cytometry

**Figure 1:**
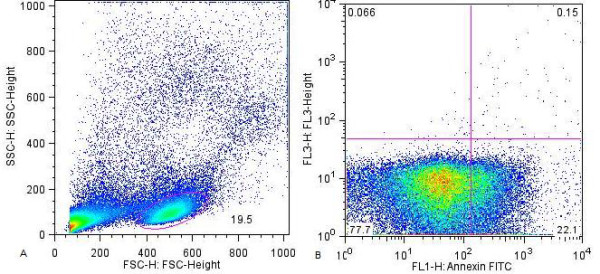
**Flow cytometry scatter plots for the Annexin-V assay**. A: PBL were identified and gated based on forward and side scatter properties. B: Scatter plots for green and red fluorescence channels were used to measure Annexin-V and PI positivity respectively.

PBL were identified and sorted according to forward and side angle scatter morphological parameters (Figure 1A). Labeled PBL in the assays above were enumerated by flow cytometry using a 4-colour FACS Calibur (BD Biosciences, Belgium) flow cytometer. Data was acquired with CellQuest Pro software (BD Biosciences, Belgium) from 100, 000 events for each assay. Analysis was performed with FlowJo 7.1 software (Tree Star Inc., USA).

### Apoptotic protease activity

Luminometry assays determined the activities of apoptotic initiator caspase 9 and executioner caspases 3/7. Separate aliquots of PBL (1 × 10^5^) were transferred into luminometry-quality white microtitre plates. 100 μl of caspase substrate (Caspase-Glo 3/7, Caspase-Glo 9; Promega, USA) was added to PBL and allowed to react for 30 minutes. Luminescent signals were then measured with the Modulus microplate luminometer (Turner Biosystems, USA) and expressed as relative light units.

### Analysis of apoptotic pathways by quantitative PCR (qPCR) array

RNA was extracted and purified from PBLs using Trizol reagent (Invitrogen, USA) as per manufacturer's instructions and thereafter quantified by spectrophotometry. Expression of 84 critical genes which represent 10 functional gene groups involved in apoptotic signal transduction pathways were analyzed by quantitative PCR using the Human Apoptosis RT^2 ^Profiler PCR array (SABiosciences, USA), according to the manufacturer's protocol. Details regarding the technology and specific genes can be found at the following URL: http://www.sabiosciences.com/rt_pcr_product/HTML/PAHS-012A.html.

Briefly, cDNA was prepared from 1 μg of total RNA using the RT^2 ^PCR array first strand kit (SABiosciences, USA). qPCR reactions were conducted in a 25 μl mixture which included 12.5 μl of 2X qPCR master mix, 11.5 μl of nuclease free water and 1 μl of cDNA template. The thermal cycle profile consisted of an initial 10 minute step at 95°C followed by 40 cycles of 95°C for 15 seconds and 60°C for 1 minute. Real time quantitations were carried out using the Bio-Rad Chromo4 real time detection system (Bio-Rad, USA). Data was analyzed using SABiosciences web-based qPCR data analysis service. Fold changes were calculated using the 2^- Δ ΔCt ^method.

### Detection of HSP70 by Western blot

Total PBL protein was extracted from each sample using Cytobuster™ (Calbiochem, UK) reagent, supplemented with protease inhibitors, as per manufacturer guidelines. Protein concentration was determined by the bicinchoninic acid assay (Sigma, Germany) and standardized to 250 μg/ml. Samples containing 10 μg of protein were boiled in Laemmli buffer for 5 minutes and then subjected to electrophoresis in 10% sodium dodecyl sulfate (SDS)-polyacrylamide gels. Separated proteins were then electro-transfered to polyvinylidene difluoride membranes (PVDF). After blocking with Tris-buffered saline (TBS) containing 5% nonfat dry milk and 0.1% Tween 20, the membrane was immuno-probed with monoclonal anti-HSP70 (1:5, 000; Pharmingen, USA) for 1 hour at RT. The PVDF membrane was then subjected to 5 washes (10 minutes each) with TBS containing 0.1% Tween 20. The membrane was then exposed to secondary antibody (anti-mouse-horse-radish-peroxidase (HRP)-conjugate; 1:10, 000; Bio-Rad, USA) for 1 hour at RT. Anti-β-actin-HRP (Sigma, Germany) was utilized for internal loading controls. After further washing, antigen-antibody complexes were detected by chemiluminescence using the Immune-star™ HRP substrate kit (Bio-Rad, USA). Chemiluminescent signals were detected with the Chemi-doc XRS gel documentation system. Images were acquired and analyzed with Quantity-one™ image analysis software (Bio-Rad, USA). Data is represented as peak band intensity for each sample.

### DNA fragmentation assay

Genomic DNA was extracted from PBL (1 × 10^5^) for each sample. Cells were transferred to 500 μl lysis buffer containing 0.5% SDS, 150 mM NaCl, 10 mM EDTA, and 10 mM Tris-HCl (pH 8.0). To this RNase A (100 μg/ml; DNase-free) was added and the solution was incubated at 37°C for 1 hour. Subsequently proteinase K (200 μg/ml) was added to the solution and incubated for a further 3 hours at 50°C. Protein contaminants were then precipitated by addition of 0.1 volume 5 mM potassium acetate and centrifugation (5, 000 × g; 15 minutes). Supernatants containing genomic DNA were transferred to fresh tubes and extracted with 100% isopropanol on ice, and thereafter washed with 70% ethanol. DNA samples were then dissolved in 10 mM Tris and 0.1 mM EDTA (pH 7.4) at 4°C overnight. Concentration of each sample was determined spectrophotometrically. To prepare a positive control for the DNA fragmentation assay, apoptosis was induced in control PBL samples by treating with camptothecin (4 μg/ml, 12 hours) *in vitro*. DNA was extracted and quantified as described above. Equal amounts of DNA (300ng) were electrophoresed (150V; 50 minutes) on a 1.8% agarose gel containing 0.5 mg/ml ethidium bromide. DNA bands were visualized by UV light and digitally photographed using a gel documentation system and Quantity-one™ image analysis software (Bio-Rad, USA). Quantity-one™ software was used to quantify the density of bands for comparison between experimental groups.

### Statistical analysis

Non-parametric Mann-Whitney tests were used to test for statistical differences between patients and controls for all apoptosis assays. Pearson, or Spearman rank correlations were used to test the dependence of variables where appropriate. All analyses were performed using the GraphPad Prism (V5) software package (GraphPad Software Inc., USA).

## Results

### Elevated phosphatidylserine externalization in RA-PBL

Translocation of phosphatidylserine residues from the inner leaflet of the plasma membrane to the outer leaflet of the plasma membrane is an early apoptotic event. Annexin-V is a specific and strong phosphatidylserine-binding protein [24] that detects cells undergoing apoptosis. The annexin-V assay showed that apoptosis was significantly higher (p < 0.05) in RA PBL than in healthy controls *ex vivo*. When analyzed separately, apoptosis in RA CD4^+ ^PBL was approximately 3.5-fold higher than controls (26.3 ± 1.6% vs. 7.6 ± 0.8%). The highest apoptosis values were recorded in the RA CD19^+ ^PBL, which were approximately 4-fold higher than controls (60.5 ± 7.4% vs. 16.1 ± 1.9%; Table 2).

**Table 2:** Annexin-V analysis of apoptosis in peripheral lymphocyte sub-populations from South African rheumatoid arthritis patients and race-matched controls *ex vivo*

Cell population	Patients *[Mean % (SEM)]*	Controls *[Mean % (SEM)]*
Total PBL	30.0 (1.5)*	7.2 (0.9)
CD4^+ ^PBL	26.3 (1.6)*	7.6 (0.8)
CD19^+ ^PBL	60.5 (7.4)*	16.1 (1.9)

* Significant difference (p < 0.05; Mann-Whitney test). CD4, cytotoxic T-lymphocyte marker; CD19, pan B-lymphocyte marker.

Late stage apoptotic and/or necrotic cells which take up PI were distinguished from PBL which were exclusively positive for annexin-V (Figure 1). The percent of RA-PBL positive for PI was extremely low (0.4% ± 0.10) and did not differ significantly from controls (0.2 ± 0.03; p = 0.1085, unpaired t test).

**Figure 2:**
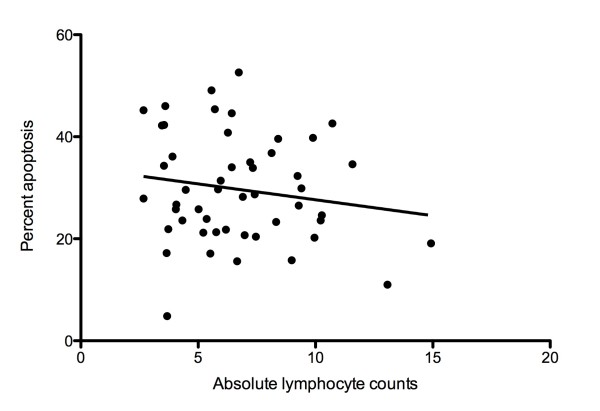
**Absolute lymphocyte counts did not correlate with peripheral lymphocyte apoptosis in RA patients**. Correlation co-efficient *r *= -0.08956; *p *value = 0.5362; Pearson *r *correlation.

To examine the effect of elevated apoptosis on the number of circulating lymphocytes in RA patients, we statistically tested whether total PBL apoptosis correlated with absolute lymphocyte counts. We found no statistically significant relationship between total PBL apoptosis and absolute lymphocyte counts (Figure 2).

In order to determine whether disease duration affected apoptosis levels in our study cohort, we examined whether apoptosis correlated with disease duration. We found that levels of apoptosis did not correlate with disease duration (Spearman rank correlation, r = -0.1539, p = 0.2962). Patients were then grouped, i.e. patients with RA for < 10 years (n = 22) and > 10 years (n = 28). Although total PBL apoptosis was slightly higher in patients with RA for less than 10 years (30.6% vs. 27.8%), disease duration did not significantly affect apoptosis in our patient cohort (*p *= 0.6843; unpaired t test with Welch correction).

**Figure 3:**
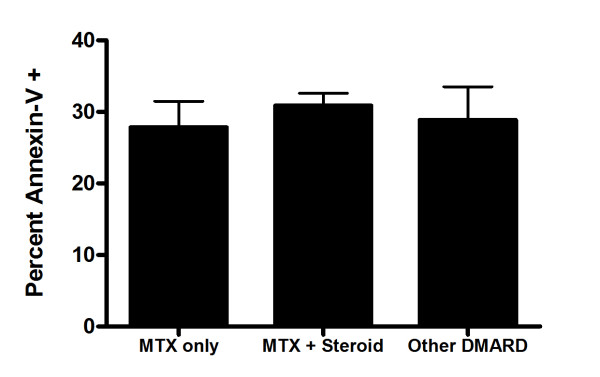
**Percent of peripheral blood lymphocytes with translocated phosphatidyl serine according to treatment in RA patients (MTX: methotrexate; DMARD: disease modifying anti-rheumatic drug)**.

Patients were at various stages of treatment when sampled. They were distributed among three treatment categories, namely, patients on methotrexate (MTX) alone (n = 13), MTX together with steroidal drugs (n = 29) and those on other disease-modifying-anti-rheumatic-drugs (DMARD, n = 8). There was no significant difference in PBL apoptosis (*p *= 0.6967, one way ANOVA; Figure 3) when compared across different treatments. Experimental values measured in our patient cohort were independent of treatment.

### Higher percentage of RA-PBL with CD95/Fas on plasma membrane

**Figure 4:**
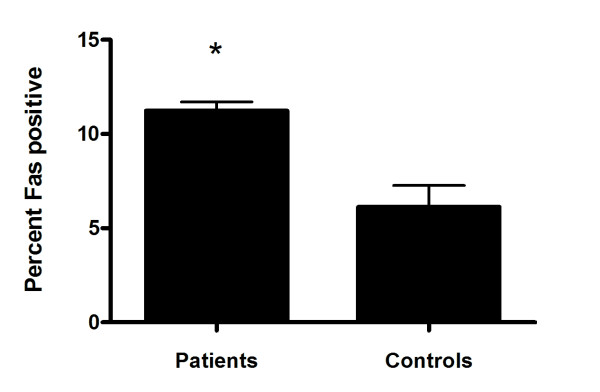
**Percent CD95/Fas positive peripheral lymphocytes from South African rheumatoid arthritis patients and healthy race-matched controls**. PBL were analyzed by flow cytometry. Data is represented as mean percent + standard error of the mean. * Significant difference, p = 0.0317; Mann Whitney test.

To determine whether the elevated apoptosis measured in our patient cohort was associated with receptor mediated apoptosis-inducing signals, we tested for the presence of CD95/Fas on PBL. The proportion of PBL expressing CD95/Fas was significantly higher in RA patients compared to controls (*p *= 0.0317; Mann Whitney test; Figure 4).

### Increased expression of Fas pathway associated transcripts in RA-PBLs

**Figure 5:**
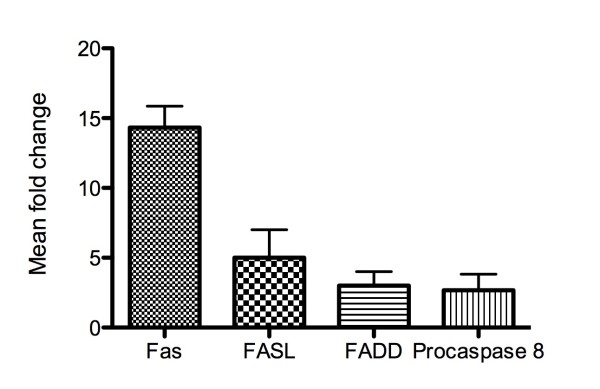
**Increased expression of Fas pathway associated genes in RA-PBL**. Data is represented as mean fold change + standard error of the mean.

To investigate apoptotic signaling pathways in RA-PBLs, we analyzed the expression profiles of 84 genes involved in the induction and/or transduction of apoptosis. The highest fold change differences were observed in genes associated with the Fas-mediated apoptotic pathway (Figure 5).

### RA-PBL showed low levels of activation

**Figure 6:**
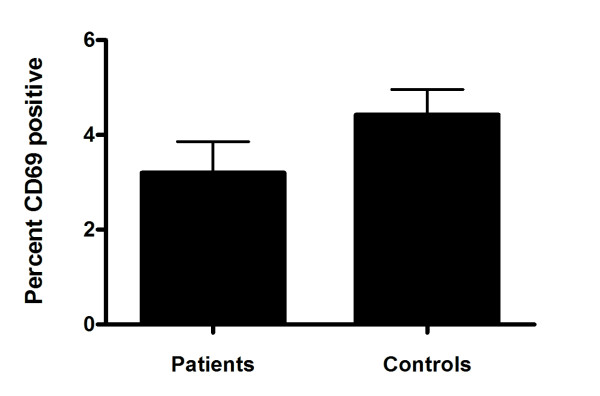
**Activation status of peripheral lymphocytes were monitored by examining presence of CD69 on cell surface**. Data is represented as mean percent + standard error of the mean. No statistical significance was established between RA patients and control subjects (p > 0.05; Mann Whitney test).

Since CD95/Fas is associated with AICD in lymphocytes, we examined the activation status of circulating lymphocytes in our study cohort by monitoring the proportion of PBL positive for the CD69 activation marker. Interestingly, despite high apoptosis levels, RA patients had a lower percent of PBL positive for CD69 compared to controls, however, this did not reach statistical significance (*p *> 0.05; Mann Whitney test; Figure 6).

### Elevated caspase activity in RA-PBL

**Figure 7:**
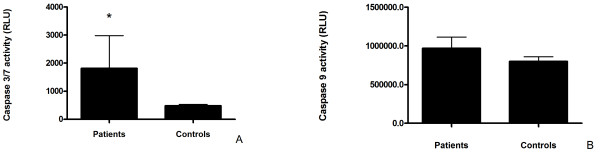
**Apoptotic protease activity in peripheral lymphocytes**. A: Higher caspase 3/7 activity in RA-PBL despite low luminescent signals. B: Initiator caspase 9 activity was high in both study groups but not significantly different. Data is expressed as mean relative light units + standard error of the mean. * Significant difference, p < 0.01; Mann-Whitney test.

Despite low luminescent signals recorded for executioner caspase 3/7 activity, there was approximately 3-fold higher activity in RA PBL compared to healthy controls. This difference in activity reached statistical significance (*p *< 0.01; Mann-Whitney test; Figure 7A). Caspase-9 activity produced strong luminescent signals in both patients and controls, but was not statistically significant between these groups (Figure 7B).

### Elevated HSP70 in RA-PBL

**Figure 8:**
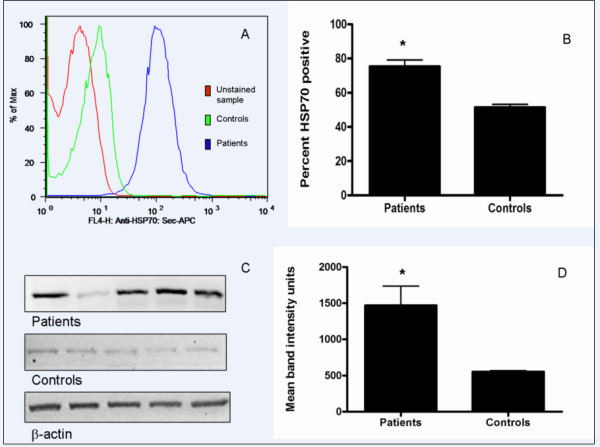
**Detection of HSP70 in peripheral lymphocytes**. A: Mean fluorescence intensity histogram for intra-cellular detection of HSP70 by flow cytometry. B: Proportion of PBL with detectable levels of HSP70 was higher in RA patients (* p = 0.0001; Mann-Whitney test). C: Representative western blot analysis of HSP70 in total PBL protein. D: HSP70 levels significantly elevated in RA patients (* p = 0.0090; unpaired t-test).

**Figure 9:**
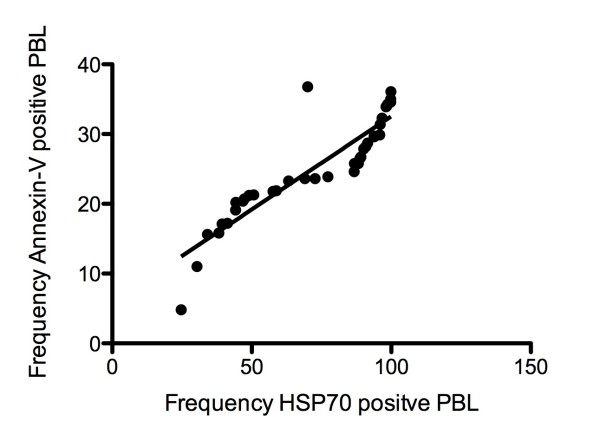
**HSP70 levels correlate with RA-PBL apoptosis**. Correlation co-efficient *r *= -0.8121; *p *value < 0.0001; Pearson *r *correlation.

Since HSP70 is an inducible protein which can modulate apoptosis signals, the levels of HSP70 in PBL were examined. Using intra-cellular flow cytometry, we distinguished between PBL with high or low levels of intra-cellular HSP70 as a function of mean fluorescence intensity (Figure 8A). Our data showed that the proportion of PBL with detectable levels of intra-cellular HSP70 was significantly higher in RA patients compared to controls (*p *= 0.0001; Mann-Whitney test; Figure 8B). To confirm these data, western blot analysis for HSP70 was performed on total PBL protein (Figure 8C). Band analyses showed that HSP70 levels were significantly elevated in RA patients (*p *= 0.0090; unpaired t-test; Figure 8D). We next investigated whether there was an association between HSP70 levels and phosphatidylserine externalization in RA-PBLs. Our data showed a strong positive correlation between HSP70 levels and phosphatdylserine externalization in RA-PBLs (Figure 9).

### No DNA fragmentation in RA-PBL

**Figure 10:**
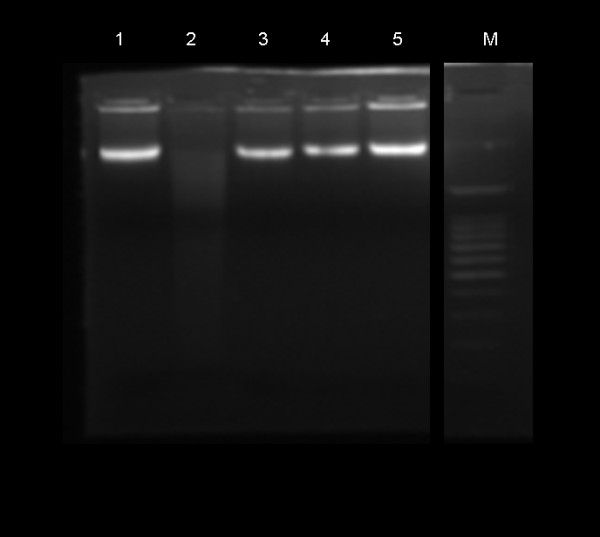
**DNA fragmentation assay in peripheral lymphocytes by agarose gel electrophoresis**. M: molecular weight marker; lane 1: control PBL DNA; lane 2: positive control; lane 3, 4, 5: RA-PBL DNA.

DNA fragmentation, a typical molecular feature of apoptosis, occurs due to nuclease mediated cleavage of genomic DNA into oligonucleosomal fragments in multiples of approximately 200 base pairs. Nuclease activity is induced towards the latter stages of apoptosis by upstream apoptosis mediators such as caspase-3. The characteristic apoptosis DNA fragmentation pattern was not observed in patient or control samples following agarose gel electrophoresis. All DNA bands were of high molecular weight and intact with no signs of apoptosis-induced DNA damage (Figure 10).

## Discussion

The fine balance between cell survival and cell death is essential for homeostasis in multi-cellular organisms. Apoptosis is the major mechanism of physiological cell death which facilitates deletion of unwanted or damaged cells. It plays a central role in the immune system in both the maintenance of self-tolerance and homeostatic control of lymphocyte populations [4]. The immune system relies on apoptosis for its functional integrity at multiple levels, and consequently, stringent regulation of these pathways is imperative. Immunological tolerance is promoted by carefully directed apoptosis in self-reactive T-lymphocyte clones during their maturation in the thymus [25]. Immune learning continues while lymphocytes are in peripheral circulation, since not all antigenic combinations are encountered in the thymus. The precise mechanisms of peripheral immune learning are unknown, but may also involve deletion of self-reactive lymphocytes by apoptosis whilst in circulation [26]. It is likely that defects in peripheral immune learning may lead to autoimmunity. Lymphocyte death is tightly regulated and there are detrimental consequences when regulatory mechanisms are compromised. For instance, abnormal increases in apoptosis can cause immunodeficiency [27]; while a failure to undergo apoptosis can lead to development of autoimmunity [28].

In RA, there is compelling evidence to show that a compromise in lymphocyte apoptosis contributes to the persistence of these cells at inflamed joints [6]. Inflammation is normally resolved by carefully directed apoptosis of invading immune cells [4]. In RA however, the molecular interactions between synovial cells and infiltrating lymphocytes have been shown to protect against apoptosis in the synovium [8].

Little is known about the biological status of circulating lymphocytes prior to synovial recruitment in RA. Our studies therefore focused on circulating lymphocytes, which perpetuate the autoimmune manifestations of RA. We assessed PBL apoptosis since they are exposed to a myriad of pro-inflammatory cytokines and acute-phase proteins. This may compromise functional integrity before the PBL adopt a stationary phenotype in the rheumatoid synovium. Our data showed that early markers of apoptosis were elevated in total lymphocytes from RA patients. This trend was mimicked in CD4^+ ^lymphocytes, but more so in CD19^+ ^B-lymphocytes; where more than half of this lymphocyte population showed apoptotic features whilst in circulation. This suggests that abnormalities in the regulation of lymphocyte apoptosis may occur early in RA, prior to synovial infiltration.

Our data also showed minor elevations in control PBL apoptosis compared to baseline values previously reported in the literature. This may be indicative of normal baseline fluctuations in apoptosis levels since lymphocytes are extremely sensitive to physiological changes. Considering baseline apoptotic noise, it is likely that observations of relative apoptotic changes, which deviate significantly in disease phenotypes, may in fact indicate an integral role for the apoptosis in disease progression or etiology.

The elevated levels of PBL apoptosis observed in our patient cohort was not associated with AICD, since only a small percentage of lymphocytes showed detectable levels of the CD69 activation marker. CD69 is a transiently expressed membrane receptor early during lymphocyte activation, but is also selectively expressed in chronic inflammation [29]. Interestingly, engagement of the CD69 receptor was shown to trigger apoptosis in multiple cell types [30], but despite persistent expression in chronic inflammatory infiltrates, lymphocyte apoptosis was inhibited. Evidence from molecular and cellular studies showed that T-lymphocyte activation was altered in RA [31]. This may account for the high levels of RA-PBL apoptosis observed in our patients where signs of early lymphocyte activation were relatively absent.

Under normal conditions, healthy mitochondria have polarized electronegative transmembrane gradients due to oxidative phosphorylation reactions. We have previously reported that mitochondrial depolarization was elevated in RA-PBL [32]. Loss of transmembrane potential alters mitochondrial permeability which results in the release of proteins such as cytochrome *c *and second-mitochondrial activator of caspases/direct-inhibitor-of-apoptosis-binding-protein-with-low-PI (Smac/DIABLO) into the cytoplasm [33]. In the cytoplasm, cytochrome *c *binds to apoptotic-protease-activating-factor (Apaf-1) and pro-caspases, leading to ATP-dependant formation of the apoptosome [34]. The apoptosome is a potent activator of initiator caspases, in particular caspase-9. Activated caspase-9 in turn facilitates activation of executioner caspases (primarily caspase-3 and caspase-7), which co-ordinate proteolytic breakdown of apoptotic cells. Engagement of CD95/Fas with its ligand leads to activation of caspase-8 following recruitment of Fas-associated-death-domain-protein (FADD). This can signal apoptosis via two well-described pathways: (i) direct activation of caspase-3; or (ii) alteration of mitochondrial transmembrane potential via Bcl-2 homology-3 (BH3)-interacting-death-domain (BID) agonist, which initiates the mitochondrial apoptosis cascade [35,36]. The CD95/Fas signaling pathway ultimately culminates in the activation of executioner caspases, which is a molecular hallmark of apoptosis.

Although caspase-3/7 activity was significantly higher in RA patients, these activities remained relatively low. This suggests that there may be perturbations in the signaling pathways which activate executioner caspases in these cells. A possible mechanism may involve HSP mediated interference between apoptosis initiator signals and their down-stream targets.. Activation of caspase-3 for instance, is suppressed by HSP27 since it binds to pro-caspase-3, thus preventing its activation by caspase-9 [37]. Alternatively, HSP27 may sequester cytochrome *c *from Apaf-1, thus preventing assembly of the apoptosome [38,39]. In addition the small HSP αβ crystalline, suppresses cytochrome *c*-mediated autoactivation of caspase-3, by direct interaction with the enzyme to prevent its complete processing [40]. HSP70 has been implicated in the inhibition of apoptosome formation [20,41], but may also inhibit caspase-dependent events that occur later in apoptosis [42]. Chromosomal DNA is digested by caspase-activated-DNase/DNA fragmentation factor 40 (CAD/DFF40) during the final stages of apoptosis, upon activation by caspase-3 [43]. The enzymatic activity and structural integrity of CAD/DFF40 was reported to be regulated by HSP70 and HSP40 [44]. Over-expression of these HSPs may prevent nuclear degradation regardless of up-stream pro-apoptotic events. We have recently reported in the same population of patients that RA-PBL sustain significant damage due to oxidative stress [32]. This may induce cellular stress responses which increase the expression of HSPs, which could possibly modulate apoptotic signal cascades. This may have contributed to the lack of lymphocyte DNA fragmentation observed in our patient cohort, despite early signs of apoptosis. Earlier studies by Szodoray *et al *(2003) examined nuclear condensation as a measure of apoptosis in circulating RA T-lymphocytes bearing typical apoptotic markers (CD95/Fas, Bax, Bcl-2 and TNF receptor) [45]. These investigations showed decreased levels of nuclear condensation in T-lymphocytes and were thus interpreted to have decreased rates of CD95/Fas mediated apoptosis. In addition, lymphocytes positive for Bax protein also showed decreased apoptosis frequency. The study concluded that the reduced susceptibility to CD95-mediated apoptosis may contribute to the expansion of an activated CD4^+ ^lymphocyte sub-population and thus to the maintenance of peripheral autoreactive T-cell clones in RA [45].

Furthermore, the molecular features of apoptosis measured in RA-PBL did not translate to reduced numbers of circulating lymphocytes in our patient cohort. This was indicated by a lack of statistically significant correlation between absolute lymphocyte counts and total PBL apoptosis. This observation supports the notion that the apoptotic program may not be fully executed despite early molecular signs of apoptosis in RA-PBL. Albeck *et al *(2008) recently reported that although cells may exhibit molecular hallmarks of apoptosis, they may not be committed to fully executing the program and may recover from pro-apoptotic signals [46]. Although the mechanisms of apoptosis recovery are not fully understood, caspase inhibition via the X-linked-inhibitor-of-apoptosis-protein (XIAP) and proteosomal degradation of executioner caspases seem to play a role [47]. Interestingly, Rehm *et al *(2006) also described a state in which cells may exist with partial caspase-dependent degradation of their proteomes without outward manifestations of apoptotic features [48]. In RA, it is likely that although apoptosis is initiated in circulating lymphocytes, these cells may not be committed to executing the molecular program fully and cellular interactions at the synovium exacerbate their anti-apoptotic phenotype. In addition, chronically elevated lymphocyte counts may occur as a result of apoptosis induced compensatory proliferation. Recent studies have elucidated non-apoptotic functions of both initiator and executioner caspases. These are involved in generating growth stimulating and compensatory cell proliferation signals via alternate MAPK cascades [49]. Death receptors have also been implicated in non-cytotoxic responses which include regulation of cell proliferation, growth stimulation and production of pro-inflammatory chemokines. Evidence already indicates that engagement of death receptors in the rheumatoid synovium promotes cell proliferation instead of cell death [50].

Although not fully understood, similar mechanisms may operate and contribute to the maintenance of autoreactive lymphocyte clones in autoimmune diseases where apoptosis is elevated in peripheral circulation.

## Competing interests

The authors declare that they have no competing interests.

## Authors' contributions

DM conceived the study, conducted all experimental laboratory experiments, analysed data and prepared the draft manuscript. GM participated in the design of the study and preparation of the final draft. AC participated in the design of the study, data analysis and preparation of the final draft. All authors read and approved the final manuscript.
